# The anti-tumor activator sMEK1 and paclitaxel additively decrease expression of HIF-1α and VEGF via mTORC1-S6K/4E-BP-dependent signaling pathways

**DOI:** 10.18632/oncotarget.2119

**Published:** 2014-06-19

**Authors:** Boh-Ram Kim, Kyungsil Yoon, Hyun-Jung Byun, Seung Hee Seo, Seung-Hoon Lee, Seung Bae Rho

**Affiliations:** ^1^ Research Institute, National Cancer Center, 323, Ilsan-ro, Ilsandong-gu, Goyang-si Gyeonggi-do, Republic of Korea; ^2^ Department of Life Science, Yong In University, 470, Samga-dong, Cheoin-gu, Yongin-si Gyeonggi-do, Republic of Korea

**Keywords:** sMEK1 anti-activator, cell cycle arrest, caspase activity, traditional chemotherapeutic agent, ovarian cancer

## Abstract

Recently, we found that sMEK1 effectively regulates pro-apoptotic activity when combined with a traditional chemotherapeutic drug. Therefore, combinational therapeutic strategies targeting critical molecular and cellular mechanisms are urgently required. In this present work, we evaluated whether sMEK1 enhanced the pro-apoptotic activity of chemotherapeutic drugs in ovarian carcinoma cells. Combined with a chemotherapeutic drug, sMEK1 showed an additive effect on the suppression of ovarian cancer cell growth by inducing cell cycle arrest and apoptosis and regulating related gene expression levels or protein activities. In addition, the phosphoinositide-3-kinase (PI3K)/Akt/mammalian target of rapamycin (mTOR) pathway was strongly inhibited by the combined treatment, showing de-repression of the tuberous sclerosis complex (TSC) and suppression of ras homolog enriched in the brain (Rheb) and mTOR and raptor in aggressive ovarian carcinoma cells and mouse xenograft models. Treatment with sMEK1 and paclitaxel reduced phosphorylation of ribosomal S6 kinase (S6K) and 4E-binding protein (4E-BP), two critical downstream targets of the mTOR-signaling pathway. Furthermore, both sMEK1 and paclitaxel significantly inhibited the expression of signaling components downstream of S6K/4E-BP, such as hypoxia-inducible factor-1α (HIF-1α) and vascular endothelial growth factor (VEGF), both *in vitro* and *in vivo*. Therefore, our data suggest that the combination of sMEK1 and paclitaxel is a promising and effective targeted therapy for chemotherapy-resistant or recurrent ovarian cancers.

## INTRODUCTION

Ovarian cancer shows the highest mortality rates of all malignant gynecologic tumors, and its prognosis is very poor. Thus, new and effective combinational forms of therapy that target specific signaling pathways are required. Chemotherapy is one of the most widely used treatments for tumor patients. For example, chemotherapy can generally be used in addition to other modalities, such as surgery or radiation therapy. Paclitaxel is a powerful chemotherapeutic drug that binds to microtubules and prevents division in malignant ovarian, lung, breast, and prostate tumor cells [1, 2]. It also acts through the induction of G_2_/M cell cycle arrest, with subsequent mitotic arrest and apoptosis [3]. In addition, paclitaxel is considered one of the most clinically active chemotherapeutic drugs for the treatment of a variety of solid tumors, including gynecologic cancers [4, 5]. However, its effects are limited by drug resistance. Several biological functions/mechanisms of drug resistance in tumors have been postulated, including high expression of multidrug-resistance (MDR) proteins, inhibition of apoptotic signaling pathways, and enhanced DNA repair [6-8]. Apoptotic cell death is generally characterized by chromatin condensation, DNA fragmentation, and cell shrinkage. Apoptosis is an important mechanism of drug-stimulated cancer cell death, with the sensitivity to apoptosis of the various types of carcinoma cell being a major determinant of chemotherapy and radiation efficacy. Paclitaxel treatment itself can regulate the expression and mediate post-translational modifications of the Bcl-2 family proteins [9-12].

The mammalian target of rapamycin (mTOR) is a major component of the PI3K/Akt signaling pathway that is frequently dysregulated in various types of cancer, including ovarian cancer. Disordered mTOR activity has been reported to be associated with some malignant and resistant cancers. Targeting the mTOR signaling pathway could represent an effective tumor treatment strategy [13]. The mTOR kinase forms two distinct functional complexes known as mTOR complex 1 (mTORC1) and mTOR complex 2 (mTORC2), which regulate cell proliferation, metabolism, apoptosis, autophagy, and protein synthesis. mTORC1 responds to multiple signaling inputs by modulating its upstream regulators, such as tuberous sclerosis complex 1 and 2 (TSC1/2), and by direct phosphorylation of the mTORC1 component, Raptor [14-16]. In the mTOR-signaling pathway, ras homolog enriched in the brain (Rheb) guanosine triphosphatase (GTPase) are major regulators of mTORC1 activity. Upon activation, mTORC1 phosphorylates a number of proteins involved in protein biosynthesis, including ribosomal protein S6 kinases 1 and 2 (S6K1/2) and the eukaryotic initiation factor 4E-binding proteins (4E-BPs) [17]. In addition, mTORC2 plays an important role in organization of the actin cytoskeleton [14]. These effects are modulated by the mTORC2 dependent phosphorylation of protein kinase family members, such as protein kinase B (PKB) and protein kinase C (PKC).

sMEK1 tumor suppressor, termed the protein phosphatase 4 regulatory subunit 3 (PP4R3), is a highly conserved protein phosphatase family of serine/threonine phosphatases associated with sensitivity to traditional anti-cancer drugs [18, 19]. sMEK1 plays an important role in cellular biological functions, such as microtubule organization, apoptosis, cell cycle arrest, growth, DNA damage checkpoint, TNF signaling, and PI3K/Akt signaling [20-22]. sMEK1 interacts with various intracellular proteins, such as BLU tumor suppressor [23], histone deacetylase 3 (HDAC3) [24], the insulin receptor substrate 4 (IRS-4) [25], and the targets of rapamycin (TOR) [26]. Recently, Byun et al., [22] reported that sMEK1 could effectively regulate the pro-apoptotic activity of gemcitabine by upregulating p53 expression. In addition, the expression of sMEK1 was significantly suppressed in ovarian and cervical tumor patients, as well as tumor cell lines, while also being hypermethylated [23].

In this report, we explored whether sMEK1 sensitizes cancer cells to paclitaxel-induced cell death and assessed the underlying biological mechanisms. sMEK1 co-treatment could further increase paclitaxel-induced cancer cell death by enhancing apoptosis and inhibiting the mTOR-S6K/4E-BP signaling pathways more effectively. Furthermore, the expression levels of HIF-1α and VEGF, which are located downstream of mTOR, were decreased markedly. Our results suggest that sMEK1 has a novel biological function; i.e., further activation of paclitaxel-stimulated apoptosis via the concomitant inhibition of the Akt-Rheb and mTORC1-S6K/4E-BP signaling pathways.

## RESULTS

### Treatment with sMEK1 plus a chemotherapeutic drug significantly enhances cytotoxicity to ovarian carcinoma cells

**Figure 1:**
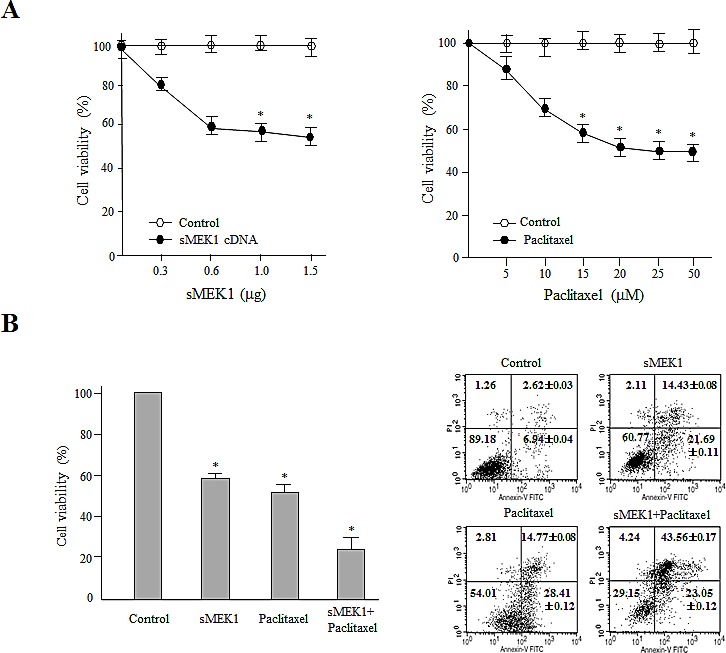
Combination treatment of sMEK1 and a chemotherapeutic drug significantly enhances cytotoxicity to carcinoma cells (A) Exponentially growing cells were treated with increasing concentrations of sMEK1 (0–1.5 μg) or paclitaxel (0–50 μM) and the effects on growth were evaluated by MTT assay. Results are expressed as means ± SD and are representative of data obtained from three independent experiments. (B) OVCAR-3 cells were treated with sMEK1 or paclitaxel alone or in combination, and cell viability was measured (left panel). In addition, the data confirmed the additive effects of sMEK1 in combination with paclitaxel using a fluorescence-activated cell sorting (FACS) system (right panel). Each data point represents the mean of triplicate samples and the bars are ± SD. *P<0.05. The experiments were repeated three times with similar results.

To examine the cytotoxic effects of sMEK1 and paclitaxel on OVCAR-3 carcinoma cells, cell proliferation was analyzed in the presence of sMEK1 (0.3–1.5 μg) or paclitaxel (5–50 μM). We also examined the combined effects of paclitaxel and sMEK1. As shown in Fig. 1A, sMEK1 and paclitaxel enhanced apoptotic cell death in a dose-dependent manner as evidenced by a ~40% reduction in cell viability by 0.6-μg sMEK1 (left panel) and 50% reduction by 20 μM paclitaxel (right panel). In order to check the combination effects, when OVCAR-3 carcinoma cells were transfected with sMEK1 (0.6 μg) and treated with paclitaxel (20 μM), cell viability was reduced by >70%, whereas a 20–30% less reduction in cell viability resulted from transfection with sMEK1 or cells treated with paclitaxel alone (Fig. 1B, left panel). In addition, we investigated the possible additive or synergistic effects of sMEK1 and paclitaxel on apoptosis using an annexin V/propidium iodide (PI)-based fluorescence-activated cell sorting (FACS) system. As shown in Fig. 1B (right panel), co-treatment with sMEK1 and paclitaxel additively decreased cell survival, suggesting that this combination could yield a greater anti-cancer effect than either treatment alone. Similar results were found in other ovarian cancer cell lines, including 2774 and SKOV-3 (Supplementary Fig. 1).

**Figure 2:**
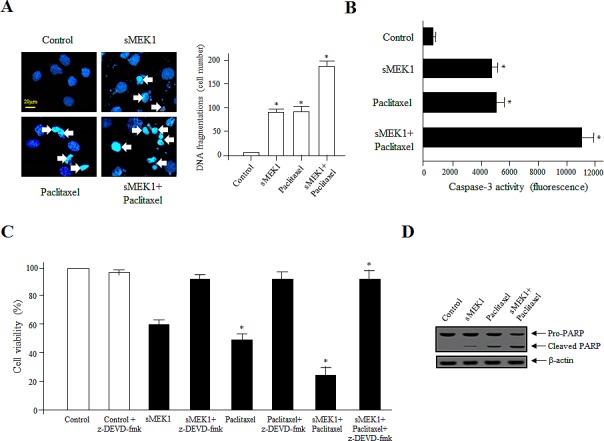
Activation of caspase-3 is important for sMEK1/paclitaxel-triggered OVCAR-3 cell apoptosis (A) Cells were treated with sMEK1 (0.6 μg), paclitaxel (20 μM), or sMEK1 plus paclitaxel followed by DAPI staining [41]. Arrows indicate DNA fragmentation. Bar: 20 μm. Data are presented as means ± SD of three independent experiments. *P<0.05 vs. the control group. (B-C) Caspase-3 activities after treatment with sMEK1, paclitaxel or both were evaluated using actyl-DEVD-7-amino-4-trifluoromethyl coumarin as the substrate. Control cells were incubated with the caspase-3-specific inhibitor, z-DEVD-fmk, and other cells were treated with sMEK1, paclitaxel, or sMEK1 plus paclitaxel. The cells were then stained with trypan blue and viability was assayed. Data are expressed as means ± SD of three independent experiments. Significant differences at the 95% confidence level (*P*<0.05) compared to the control are indicated by an asterisk (*). (D) To confirm apoptosis, cell lysates were subjected to Western blotting to evaluate proteolytic cleavage of PARP. β-actin was used to verify equal protein loading.

The effects of sMEK1 and paclitaxel on carcinoma cells were associated with the induction of apoptosis, as validated by morphological and biochemical analyses, such as DAPI staining, caspase-3 activity, and PARP cleavage. As shown in Fig. 2A, after transfection with sMEK1 or treatment with paclitaxel alone compared to the control transfectant (empty vector only), ~39–50% of the cells displayed fragmented nuclei, while no change in cell morphology was observed in control cells. Treatment with a combination of sMEK1 and paclitaxel resulted in a significantly increased number of cells harboring fragmented DNA.

Because caspase activation is an important cause of apoptotic cell death, we examined caspase-3 activity, a critical effector protease of apoptosis. Caspase-3 was activated in sMEK1-transfected and paclitaxel-treated cells compared to control cells (empty vector only). Caspase-3 activation was greater in cells subjected to sMEK1 plus paclitaxel treatment than in cells subjected to either single treatment. These results suggested that sMEK1 and paclitaxel in combination are more effective against human ovarian carcinoma (Fig. 2B). Next, cells were treated with z-DEVD-fmk, a specific inhibitor of caspase-3, for 3 h before transient sMEK1 transfection, followed by incubation for 48 h. Treatment with z-DEVD-fmk protected the sMEK1-expressing and paclitaxel-treated cells from apoptotic death. Moreover, DEVD-fmk treatment in combination with sMEK1 plus paclitaxel suppressed caspase-3 activation almost completely (Fig. 2C). Thus, ectopic sMEK1 expression additively enhanced paclitaxel-induced caspase-3 activation in carcinoma cells. We next examined the cleavage of PARP by immunoblotting. As presented in Fig. 2D, PARP cleavage was induced more strongly by co-treatment with sMEK1 and paclitaxel compared to sMEK1 or paclitaxel alone. Taken together, our results indicated that sMEK1 contributed to the anti-tumor effect by promoting paclitaxel-induced apoptotic death of cancer cells.

### Cell cycle progression and expression of cell-cycle-associated proteins in carcinoma cells

**Figure 3:**
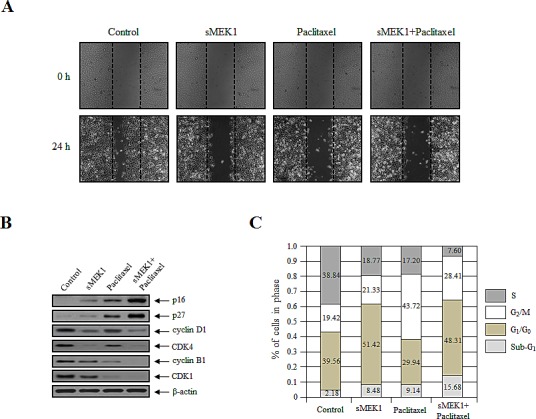
sMEK1 and paclitaxel mediate cell migration and cell cycle arrest (A) Cell migration potential was monitored using a wound-healing assay. Images of the wound areas were taken at 0 and 24 h using a Leica DFL290 camera. (B) The expression levels of sMEK1, paclitaxel or sMEK1 plus paclitaxel on cell cycle-related proteins, such as p16, p27, cyclin D1, CDK4, cyclin B1 and CDK1 in OVCAR-3 cells. β-actin served as a loading control. (C) Cell cycle progression was evaluated using FACScalibur; data are presented as characteristic DNA histograms. Cells were treated with FITC-labeled Annexin V, PI (Boehringer Mannheim, Mannheim), and RNase A (1 mg/ml) in PBS, and then incubated for 1 h at 37°C in the dark.

Wound healing is a complex and protracted process of tissue repair and remodeling in response to injury. These processes involve coordinated cell migration, cell activation and cell division. To further evaluate the effect of sMEK1 and paclitaxel on the biological processes of cancer cells, cell migration was assessed using a wound-healing assay system. A fixed-width scratch was created in a cell monolayer at 24 h post-transfection with sMEK1, paclitaxel, or sMEK1 and paclitaxel, and cell migration was monitored using a digital camera coupled to a microscope. Fig. 3A shows that at 24 h post-wound-healing, the control (empty vector only) cells migrated and almost filled the wounded area, while cells treated with sMEK1, paclitaxel, or sMEK plus paclitaxel showed significantly inhibited migration. Thus, sMEK1 or paclitaxel treatment potently suppresses cell migration *in vitro*.

To clarify the biological and molecular mechanism underlying the reduction in cell viability due to sMEK1 or paclitaxel, cell-cycle-related protein levels were examined using Western blotting. The cell cycle was then examined using a PI-based FACS analysis system in OVCAR-3 carcinoma cells treated with sMEK1, paclitaxel, or both. p16, p27, cyclin D1, CDK4, cyclin B1, and CDK1 are key regulators of cell cycle progression. Upon sMEK1 transfection alone, the expression levels of cyclin D1 and CDK4, which are associated with the transition from G_1_ to S phase, were decreased markedly compared to paclitaxel alone (Fig. 3B). Following treatment with paclitaxel alone, the expression levels of cyclin B1 and CDK1, which are associated with the transition from G_2_ to M phase, were suppressed markedly (Fig. 3B). Fig. 3B and 3C show significant reductions in cyclin D1 and cyclin B1 levels and increases in p16 and p27 levels, which are consistent with G_1_ (sMEK1 alone) or G_2_ (paclitaxel alone) phase arrest of cell cycle progression, respectively. Upon combined treatment with sMEK1 and paclitaxel, the expression levels of the CDK inhibitors p16 and p27, which are associated with the interruption of cell cycle progression in the G_1_ and G_2_/M phases, were increased markedly (Fig. 3B, C). Our findings indicate that sMEK1 and paclitaxel additively inhibit cell proliferation by inducing G_1_ or G_2_ phase arrest.

### Effect of sMEK1 and paclitaxel on the expression of apoptosis-associated proteins, and the regulation of the Bcl-2 family and NF-κB promoter activity

**Figure 4:**
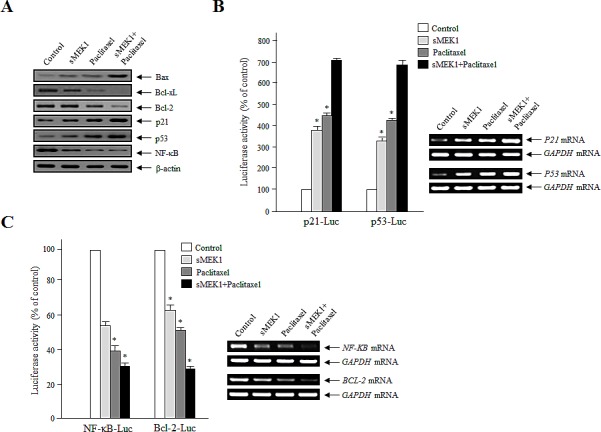
Effects of sMEK1 and paclitaxel on apoptosis-regulatory proteins, and the transcriptional activity of p21, p53, NF-κB, and the Bcl-2 promoter (A) After a 24-h treatment, the cells were collected and treated with lysis buffer. Cell lysates were subjected to Western blotting using specific antibodies against Bax, Bcl-xL, Bcl-2, p21, p53, and NF-κB. β-actin was used as the loading control. (B-C) Transcriptional activities of p21, p53, NF-κB, and the Bcl-2 promoter were evaluated using a reporter assay system with p21 (p21-Luc), p53 (p53-Luc), NF-κB (NF-κB-Luc), or Bcl-2 (Bcl-2-Luc), respectively. To correct for the differences in transfection efficiencies, the luciferase activities were normalized by co-transfection with a *Renilla* luciferase plasmid vector. The experiment was repeated three times independently; means ± SD are shown. ^*^P<0.05.

The tumor suppressor p53 and the NF-κB protein are major regulators of the signaling pathways of cell proliferation and apoptotic cell death [27, 28], and are considered key mediators of the Bcl-2 proteins [29, 30]. Expression of the anti-apoptotic Bcl-2 family proteins in OVCAR-3 cells was monitored following treatment with sMEK1 or paclitaxel. As shown in Fig. 4A, Bcl-xL and Bcl-2 expression levels were decreased markedly in sMEK1 and paclitaxel-treated cells. In addition, NF-κB levels were reduced significantly. In contrast, p53 protein levels were increased considerably in sMEK1-transfected cells and paclitaxel-treated cells, as were Bax and p21 levels. In addition, the p21 and p53 promoter activities were upregulated markedly, while NF-κB and Bcl-2 activities were downregulated gradually following treatment with sMEK1, paclitaxel, and sMEK1 plus paclitaxel (Fig. 4B and C, left panel). Subsequently, we isolated RNA from independent samples and performed RT-PCR. p21 and p53 mRNA levels were increased significantly in cells treated with sMEK1, paclitaxel, and sMEK1 plus paclitaxel compared with the controls. In contrast, NF-κB and Bcl-2 mRNA levels were decreased significantly (Fig. 4B and C, right panel). Therefore, sMEK1 or paclitaxel may enhance G_1_ or G_2_ cell cycle arrest by stimulating transcription of p21.

### Disturbance of the phosphorylation of PI3K/Akt/mTOR signaling components by sMEK1 and paclitaxel

**Figure 5:**
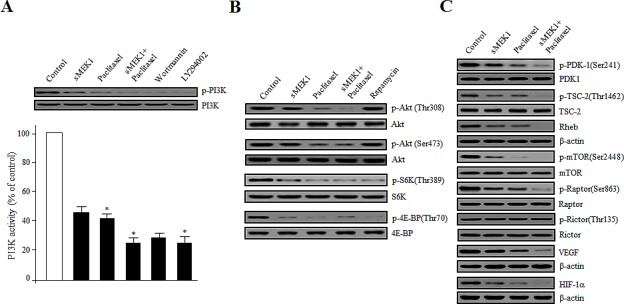
Disturbance of the phosphorylation of Akt/mTOR signaling components by sMEK1, paclitaxel, or sMEK1 plus paclitaxel (A) Effects on PI3K activity of sMEK1 and paclitaxel were evaluated using an in vitro PI3 kinase assay. Box: cells were treated with control (vector only), sMEK1 plasmid, paclitaxel, or sMEK1 plus paclitaxel, including wortmannin, or LY294002 as a PI3K inhibitor, respectively. After 24 h, cells were harvested and whole lysates were subjected to Western blotting to detect phosphorylation of the PI3K protein. PI3K was used to verify equal sample loading. The experiment was repeated three times independently; data are shown as means ± SD. *P<0.05 compared to the control (empty vector only). (B-C) After treatment with the indicated agents, protein extract samples (50 μg) were prepared, resolved by SDS-PAGE, and levels of the following proteins were determined by immunoblotting using the indicated antibodies: phospho-Akt(Thr^308^), phospho-Akt(Ser^473^), phospho-S6K(Thr^389^), phospho-4E-BP(Thr^70^), phospho-PDK-1(Ser^241^), phospho-TSC-2(Ser^1462^), phospho-mTOR(Ser^2448^), phospho-Raptor(Ser^863^), phospho-Rictor(Thr^135^), Rheb, VEGF, and HIF-1. Non-phosphorylated proteins were used as equal loading controls (indicated as Akt, S6K, 4E-BP, PDK-1, TSC-2, mTOR, Raptor, and Rictor; or β-actin for Rheb, VEGF, and HIF-1α). Three independent experiments were conducted in triplicate.

PI3Ks control numerous cellular processes, including growth, proliferation, differentiation, survival, migration, and metabolism. We first determined whether sMEK1 and paclitaxel could inhibit PI3K activity in OVCAR-3 carcinoma cells. After treatment with control (vector only), sMEK1, paclitaxel, or sMEK1 plus paclitaxel, cell lysates were immunoblotted using anti-phospho-p85 and anti-p85 antibodies, respectively. After treatment with sMEK1, paclitaxel, or sMEK1 plus paclitaxel, phosphorylation of PI3K was inhibited significantly. This inhibitory activity was comparable to that of two well-known PI3K inhibitors, wortmannin and LY294002 (Fig. 5A). PI3K inhibition in the ovarian cells resulted in inhibition of Akt phosphorylation, a major downstream target of PI3K. Akt activates mTOR through a variety of cellular mechanisms, and thus regulates apoptosis-associated proteins [31]. Generally, mTOR inhibitors play important roles in regulating the signaling pathways involved in cell cycle progression. As expected, sMEK1, paclitaxel, or sMEK1 plus paclitaxel inhibited Akt, S6K, and 4E-BP phosphorylation (Figure 5B), the latter being one of the best-characterized targets of the mTOR complex. Rapamycin was used as a positive control of inhibition of mTOR downstream signaling. These results indicated that sMEK1 and paclitaxel modulated the PI3K/Akt/mTOR pathway.

Next, we examined the involvement of phospholipid-dependent kinase-1 (PDK-1), TSC-2, Rheb, Raptor, and Rictor, which are up- or down-stream regulators of the Akt/mTOR pathway. Activated PI3K recruits PDK-1 and Akt to the plasma membrane. Akt can be phosphorylated in both a PDK-1-dependent manner and through the direct phosphorylation of mTORC2. Upon treatment with sMEK1 and paclitaxel, phosphorylation of PDK-1 on Ser241 was decreased significantly (Fig. 5C). Akt may also act indirectly on mTOR by regulating the inhibition by TSC-1/TSC-2 of mTOR activity. As shown in Fig. 5C, treatment with sMEK1 and paclitaxel decreased inhibitory phosphorylation of TSC-2 on Thr1462, which may lead to mTOR inhibition [31, 32]. As one of the key components of the PI3K pathway, mTOR exists in two different complexes; mTORC1 and mTORC2. mTORC1/Raptor phosphorylation was markedly inhibited by sMEK1 and paclitaxel, whereas that of mTORC2/Rictor was unchanged (Fig. 5C). In the mTOR signaling pathways, mTORC1/Raptor activation plays an important role in regulating translation and cell growth [33, 34]. Specifically, S6K and 4E-BP phosphorylation regulate protein synthesis, cell proliferation, and angiogenesis, including production of the VEGF and HIF-1α proteins. Treatment with both sMEK1 and paclitaxel significantly decreased VEGF and HIF-1α expression, the latter being one of the best-characterized targets of the mTOR complex in protein synthesis (Fig. 5C). Therefore, sMEK1 and paclitaxel inhibited the growth of ovarian carcinoma cells by inducing apoptotic death via the Akt-Rheb/mTORC1-S6K/4E-BP-dependent signaling pathways.

### Treatment with sMEK1 and paclitaxel inhibited the growth of human ovarian carcinoma cells in nude mice

**Figure 6:**
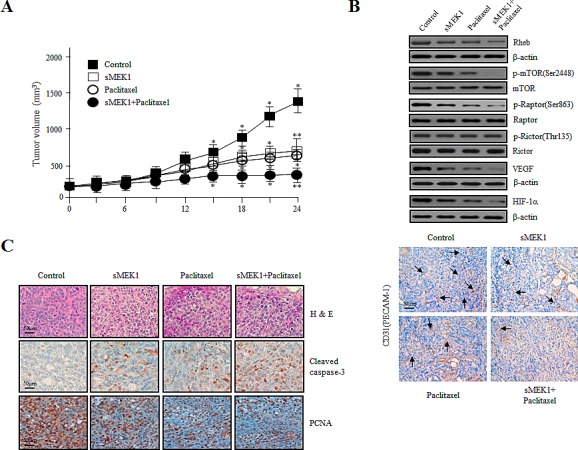
Effects of sMEK1 and paclitaxel alone or in combination on the growth of xenografts in nude mice (A) Growth curves of human ovarian xenografts treated with sMEK1, paclitaxel, and sMEK1 plus paclitaxel. Human ovarian carcinoma cells were injected subcutaneously into nude mice and allowed to grow to 70–100 mm^3^. Mice were treated by intraperitoneal injection with control, sMEK1, paclitaxel, or sMEK1 plus paclitaxel. Tumor size was measured every 3 days. *, *P*<0.05; **, *P*<0.01 compared with the control group. (B) Soluble protein extracts from xenografted mice were subjected to immunoblotting for the indicated proteins (Rheb, p-mTOR, p-Raptor, p-Rictor, VEGF and HIF-1α). mTOR, Rator, Rictor and β-actin proteins were used to verify equal sample loading. (C) Paraffin sections of sMEK-, paclitaxel-, and sMEK1 plus paclitaxel-treated and control tumors were stained with hematoxylin–eosin (H&E) (top panel). Immunohistochemical staining of the apoptotic cell death marker caspase-3 (middle panel) and cell proliferation marker PCNA (bottom panel) in xenograft mouse models. Bar: 50 μm.

The antitumor effects of treatment with sMEK1 plus paclitaxel and sMEK1 alone *in vivo* were measured in ovarian tumor xenografts. As shown in Fig. 6A, the tumor volume of the control group increased after 12 days. In contrast, tumor volumes in the sMEK1- and paclitaxel-alone groups were significantly lower than that in the control group. In addition, the combination therapy suppressed ovarian cancer tumor growth by more than 60%. Next, to confirm whether sMEK1 plus paclitaxel induced apoptotic cell death via the Akt/Rheb/mTORC1-S6K/4E-BP-dependent signaling pathways in carcinoma cells *in vivo*, ovarian-tumor-containing mice were sacrificed after each treatment and tumor tissues were subjected to Western blotting. As shown in Fig. 6B (upper panel), mTORC1/Raptor phosphorylation was suppressed markedly by sMEK1 or paclitaxel, as well as by sMEK1 plus paclitaxel, whereas that of mTORC2/Rictor was unchanged. Similar results were observed in three independent *in vitro* experiments. Subsequently, to confirm the anti-angiogenic effects of sMEK1, paclitaxel, and sMEK1 plus paclitaxel *in vivo*, we enumerated blood vessels by immunohistochemical staining for CD31 (PECAM-1). Tumor sections from sMEK1, paclitaxel, and sMEK1 plus paclitaxel-treated mice exhibited a 2.5–5 fold reduction in the number of blood vessels (Fig. 6B, lower panel). These data demonstrated that sMEK1, paclitaxel, and sMEK1 plus paclitaxel inhibited tumor angiogenesis by decreasing VEGF and HIF-1α expression *in vivo*. We next determined the anti-angiogenic effects of sMEK1, paclitaxel, and sMEK1 plus paclitaxel on VEGF-induced capillary-like tube structure formation using an *in vitro* angiogenesis model. VEGF application led to the formation of extended and strong capillary-like tubule structures, which comprised a considerably larger number of cells compared with non-VEGF-induced cells. As shown in Supplementary Fig. 2, treatment with sMEK1, paclitaxel, and sMEK1 plus paclitaxel significantly abrogated VEGF-induced tube formation. These results indicated that sMEK1 plus paclitaxel exerted a strong anti-angiogenic effect compared to either agent alone in the *in vitro* angiogenesis system.

Tumor tissue paraffin sections were visualized using H&E staining for histological analysis. The control group displayed high-grade carcinoma with an irregular cell distribution. In contrast, sections from the sMEK1, paclitaxel, and sMEK1 plus paclitaxel-treated groups exhibited large areas of late-apoptotic or necrotic cells. Subsequently, to explore whether sMEK1, paclitaxel, or sMEK1 plus paclitaxel induces apoptotic cell death, we evaluated caspase-3 activation by immunohistochemistry. As expected, sMEK1 plus paclitaxel as well as sMEK1 or paclitaxel alone significantly increased the caspase-3 activity in a xenograft tumor compared with the control group (Fig. 6C). In addition, tissues from each treatment group and the control group were stained for the cell proliferation marker PCNA. PCNA expression in the sMEK1, paclitaxel, and sMEK1 plus paclitaxel treatment groups was significantly lower than that in the control group (Fig. 6C). These results indicated that sMEK1 plus paclitaxel suppresses tumor growth by inducing apoptosis *in vivo*.

## DISCUSSION

We reported previously that sMEK1 can control apoptotic cell death by upregulating p53 expression and inhibiting Akt/mTOR [22]. The tumor suppressor sMEK1 functionally regulates apoptosis through mTOR regulation. Further studies are required to fully understand the roles of downstream components that promote death of cancer cells. In this study, the combination of the traditional chemotherapeutic paclitaxel with sMEK1 had an additive effect on inducing G_1_ or G_2_ phase arrest during cell cycle progression (Fig. 3). Our data also indicate that sMEK1 is an important modulator of mTORC1-dependent apoptotic cell death under physiological/biological conditions. The PI3K/Akt/mTOR pathway plays a critical role in apoptosis. Phosphorylation of major factors in the mTOR-signaling pathway, such as PI3K, Akt, PDK-1, and TSC-2, was decreased by treatment with paclitaxel plus sMEK1 (Fig. 5). Previous studies have shown that the inhibitory effect of TSC-1/TSC-2 on mTORC1 is modulated by TSC-2 inactivation of a Ras family small GTPase known as Rheb (Ras Homolog Enriched in Brain) [34]. Our results confirmed that the combination of these two compounds additively decreased Rheb expression (Fig. 5C). Activated mTOR regulates the phosphorylation of 4E-BP and S6K. Specifically, S6K is the best-examined target of mTORC1. S6K, which is a mitogen-activated serine/threonine kinase, is an important regulator of protein synthesis, and thus plays a key role in cell proliferation and survival [35, 36]. Shafer et al., [37] reported that paclitaxel alone suppresses phosphorylation of the S6K protein, with the greatest effect observed after 72 h of exposure, which is consistent with our results (Fig. 5B). The combination of sMEK1 and paclitaxel strongly decreased phosphorylation of the 4E-BP protein, an essential factor for protein synthesis (Fig. 5B). Hypoxia-inducible factor 1 (HIF-1) plays a critical role in cancer cell survival by regulating the transcription of various genes involved in angiogenesis, glucose metabolism and survival [38]. Walmsley et al., [39] reported that neutrophil survival is mediated by HIF-1α-dependent NF-κB mRNA expression and activity in hypoxia. Along with HIF-1α, its downstream target, VEGF, plays a key role in tumor angiogenesis and is considered an attractive chemotherapeutic target [40].

In conclusion, this study systematically analyzed the anti-tumor effects of the sMEK1 tumor suppressor protein in combination with paclitaxel, a traditional chemotherapeutic agent, using an *in vitro* and *in vivo* molecular/biochemical system. Our results characterize a detailed signaling pathway through which the combination of sMEK1 and paclitaxel induces apoptotic cell death by enhancing caspase-3 activation and PARP cleavage through the suppression of Akt-Rheb/mTORC1-S6K/4E-BP-dependent signaling pathways in ovarian tumorigenesis. In addition, this effect occurs through cellular communication with rapid inhibition of HIF-1α and VEGF expression. Importantly, our results suggest a possible molecular target for treatment with sMEK1 and paclitaxel of certain malignant tumors, which may have future clinical applications.

## MATERIALS AND METHODS

### Culture conditions, animals, chemicals, reagents and antibodies

Human ovarian carcinoma cells (OVCAR-3, MDAH-2774 and SKOV-3) obtained from the American Type Culture Collection (ATCC, Manassas, VA) were maintained in DMEM media (Life Technologies, Gaithersburg, MD) supplemented with either 10% heat-inactivated fetal bovine serum (FBS), including penicillin (100 U/ml) and streptomycin (100 μg/ml), in a humidified atmosphere containing 5% CO_2_ at 37°C. Specific pathogen-free BALB/c-nu/nu mice (5–6 weeks old) were supplied by Orientbio (Sungnam, Korea). All animal studies were approved by the Institutional Animal Care and Use Committee (IACUC) at the Research Institute of the National Cancer Center. z-DEVD-fmk caspase-3 inhibitor was purchased from Sigma (St. Louis, MO). Other traditional chemotherapeutic drugs and chemicals were obtained from Sigma. The primary antibodies used in this study were anti-sMEK1 (Abcam, Cambridge, UK), anti-pro-caspase-3, anti-cyclin D1, anti-cyclin B1, anti-CDK4, anti-CDK1, anti-Bax, anti-Bcl-xL, anti-Bcl-2, anti-p53, anti-Rheb, anti-Raptor, anti-phospho-Raptor, anti-mTOR, anti-phospho-mTOR, anti-4E-BP, and anti-phospho-4E-BP (Cell Signaling, Beverly, MA), anti-Rictor (Bethyl Laboratories, Montgomery, TX), anti-PI3K, anti-phospho-PI3K, anti-PDK-1, anti-phospho-PDK-1, anti-Akt, anti-phospho-Akt, anti-TSC-2, anti-phospho-TSC-2, anti-S6K, anti-phospho-S6K, and anti-HIF-1α (Santa Cruz Biotechnology, Santa Cruz, CA), anti-p16, anti-p27, anti-p21, and anti-NF-κB (Oncogene, San Diego, CA), anti-VEGF_121_ (Ab-1; Oncogene, Cambridge, MA), anti-PARP (BDPhamingen, San Diego, CA), anti-PCNA (Dako, Denmark), and β-actin (Sigma).

### Cell growth assay

Cell viability after treatment with sMEK1 plus paclitaxel was determined using the 3-(4,5-dimethylthiazol-2-yl)-2.5-diphenyl-^2^H-tetrazolium bromide (MTT) assay, in triplicate. Briefly, cells were plated at a density of 3.0×10^4^ per well in 96-well microtiter plates. After 24 h, cells were collected by trypsin treatment, rinsed with PBS, and replaced with fresh medium, after which 20 μl of MTT solution (Sigma, 5 mg/ml) were added to each well. Plates were then incubated for an additional 4 h at 37°C. The amounts of MTT-formazan generated were measured using a microplate reader at 570-nm absorbance. The results are representative of three independent experiments.

### DAPI staining for DNA fragmentation

OVCAR-3 cells were seeded on six-well plates and treated with control (empty vector only), sMEK1, paclitaxel, or sMEK1 plus paclitaxel. To observe their morphology, nuclei were fixed with methanol and stained for 15 min with 4, 6′-diamidino-diamidino-2-phenylindole (DAPI, 1 μg/ml, Boehringer Mannheim; Mannheim, Germany), rinsed twice with PBS, and visualized under a fluorescence microscope (Zeiss; Switzerland).

### Substrate-based caspase-3 and PARP activity analysis

Caspase activity was determined as described previously [41]. Briefly, 5.5×10^5^ cells were rinsed with cold PBS, resuspended in lysis buffer and left for 20 min on ice. The lysate was collected by centrifugation at 14,000 × *g* for 15 min at 4°C, and the supernatants were collected. Caspase-3 activity was incubated for 1 h at 37°C with specific fluorometric substrates containing z-DEVD-fmk and measured using a SpectraMax 340 microplate reader (Molecular Devices). Each individual measurement was repeated three times. PARP cleavage was assessed as described previously [42].

### Western blotting

After transfection/treatment, cells were collected, washed in PBS, centrifuged, and lysed in a buffer containing protease inhibitor (50 mM Tris, pH 7.2, 150 mM NaCl, 1% Triton X-100, 1 μg/ml leupeptin, 1 μg/ml pepstatin, 2 μg/ml aprotinin, 200 μg/ml phenylmethylsulfonyl fluoride). The cell lysates were subsequently subjected to SDS-PAGE and transferred onto Immobilon P membranes (Millipore Corporation, Billerica, MA). After blocking, the membranes were incubated with the indicated primary antibodies. The membranes were washed three times in wash buffer and incubated with horseradish peroxidase-conjugated secondary antibodies. Protein bands were detected using the ECL detection system.

### Cell migration assay

Cell migration was evaluated using the wound-healing assay as described previously [43]. Briefly, cells were seeded in 24-well plates at a density of 2.5×10^5^ per well. A scratch wound was introduced using a pipette tip. The wounded cells were washed three times with PBS and DMEM medium with 10% FBS, and covered with sMEK1, paclitaxel, or sMEK1 plus paclitaxel containing medium or the control (empty vector only) medium. Images of the wound areas were taken at 0 and 24 h using a Leica DFL290 camera and the percentage of closure was measured using the Leica application suite software (Leica Microsystems). The results are representative of three independent experiments.

### Cell cycle progression analysis and annexin V staining

Cell cycle distributions were determined by propidium iodide (PI) staining, as described previously [44]. Apoptotic cell death was assessed by staining with fluorescein isothiocyanate (FITC)-labeled annexin V and propidium iodide (PI). Briefly, cells treated with the control (expression vector only), sMEK1, paclitaxel, or sMEK1 plus paclitaxel were collected, rinsed with ice-cold PBS, and then resuspended in binding buffer. After incubation at 37°C for 1 h, the cells were incubated for 15 min with fluorescein isothiocyanate (FITC)-labeled annexin V, according to the manufacturer's protocol (Boehringer Mannheim, Mannheim), and then subjected to flow cytometry analysis (FACScalibur, Becton Dickinson, Franklin Lakes, NJ). Each individual measurement was repeated three times.

### Luciferase reporter assay

*In vitro* p21, p53, NF-κB and Bcl-2 promoter activities were evaluated as described previously [45]. Briefly, cells at 85% confluency were transfected with the indicated reporter plasmids. After lysis in RIPA buffer, lysates were cleared by centrifugation for 15 min at 14,000 rpm and cell extracts were treated with the luciferase substrate reagent at room temperature for 30 min according to the manufacturer's protocol. Then, a 5-μl aliquot of each sample was quantitated using the MicroLumat Plus LB96V luminometer.

### P21, P53, NF-κB and BCL-2 mRNA levels by reverse transcription-polymerase chain reaction (RT-PCR)

Total RNA was isolated using the TRIzol total RNA isolation system (Invitrogen). Two micrograms of total RNA were reverse transcribed into first-strand cDNA using a cDNA synthesis kit (Promega). PCR was performed in a final reaction volume of 50 μl containing specific primers synthesized by MACROGEN (Seoul, Korea) targeting the human *P21*, *P53*, *NF-κB*, and *BCL-2* genes (*P21*; forward: 5′-GCGATGGAACTTCGACTTTGT-3′ reverse: 5′-GGGCTTCCTCTTGGAGAAGAT-3′, *P53*; forward: 5′-CAGCCAAGTCTGTGACTTGCACGTAC-3′ reverse: 5′-CTATGTCGAAAAGTGTTTCTGTCATC-3′, *NF-κB*; forward: 5′-TCCGTTATGTATGTGAAGGC-3′ reverse: 5′-TTTGCTGGTCCCACATAGTTGC-3′ and *BCL-2*; forward: 5'-CGACGACTTCTCCCGCCGCTACCGC-3' reverse: 5'- CCGCATGCTGGGGCCGTACAGTTCC-3'); the human glyceraldehyde-3-phosphate dehydrogenase gene (*GAPDH*; forward: 5'-GTCAGTGGTGGACCTGACCT-3' reverse: 5'-TGAGGAGGGGAGATTCAGTG-3'). *GAPDH* was used as the internal control. After incubation at 95°C for 5 min, PCR was performed for 25 cycles of 95°C for 1 min, 56°C for 1 min, and 72°C for 1 min, and the products were visualized by agarose gel electrophoresis.

### Xenograft mouse model and immunohistochemistry

Briefly, 6-week-old female BALB/c-nu/nu mice were injected subcutaneously (s.c.) with 1.6×10^6^ OVCAR-3 ovarian carcinoma cells. When tumors reached ~100 mm^3^ in volume on day 14, mice were subjected to intraperitoneal injection of each agent, which was repeated every 3 days for 27 days. Tumor size was measured every other day in three dimensions using calipers. Mice were sacrificed 1 day after the final injection. Tumors were then excised and prepared for immunohistochemistry (IHC).

### Statistical analysis

The data are shown as means ± SD calculated using Student's *t*-test and ANOVA according to the number of groups compared. Significant differences of 95% confidence (*P*<0.05) are indicated by an asterisk (*).

## SUPPLEMENTARY MATERIALS AND METHODS FIGURES


