# Supplementary material for: The anti-tumor activator sMEK1 and paclitaxel additively decrease expression of HIF-1α and VEGF via mTORC1-S6K/4E-BP-dependent signaling pathways
Source: Oncotarget. 2014 Jun 19;5(15):6540–51. doi: 10.18632/oncotarget.2119 (PMC4171649; doi:10.18632/oncotarget.2119)
Supplement: Supplementary file 1 [file oncotarget-05-6540-s001.pdf]

# The anti-tumor activator sMEK1 and paclitaxel additively decrease expression of HIF-1α and VEGF via mTORC1-S6K/4E-BP-dependent signaling pathways

## Supplementary Materials And Methods

### *In vitro tube formation*

*In vitro* tube formation assays were performed as described previously [1, 2]. In brief, growth factor-reduced Matrigel (200 μl of 10 mg/ml) was added to a 24-well plate and polymerized at 37°C for 30 min. Control-treated, sMEK1-treated, paclitaxel-treated, or sMEK1 plus paclitaxel-treated HUVECs (2.5 $\times 10^5$ cells) were grown on the surface of the Matrigel. Seeded cells were then incubated with or without 10 ng/ml of VEGF for 48 h in M199 containing 1% FBS. After being washed, capillary-like structure images were photographed at x40 magnification. Tube formation lengths were measured using an inverted microscope equipped with a digital CCD camera (Zeiss), and quantification was used under an ImageLab imaging software (MCM Design).

## REFERENCES


1. Rho SB, Choi K, Park K, Lee JH. Inhibition of angiogenesis by the BTB domain of promyelocytic leukemia zinc finger protein. Cancer Lett. 2010; 294: 49-56.
2. Rho SB, Song YJ, Lim MC, Lee SH, Kim BR, Park SY. Programmed cell death 6 (PDCD6) inhibits angiogenesis through PI3K/mTOR/p70S6K pathway by interacting of VEGFR-2. Cell. Signal. 2012; 24: 131-139.

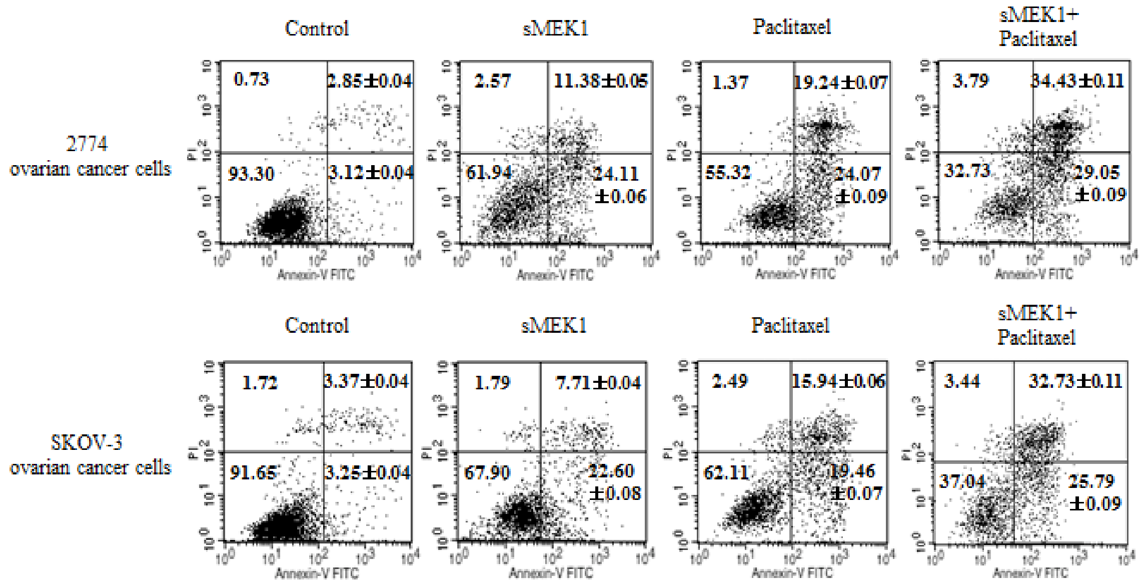


**Fig S1: Combination treatment of sMEK1 with a chemotherapeutic drug dramatically enhances the cytotoxicity to ovarian carcinoma cells.** 2774 and SKOV-3 cells were transfected/treated with sMEK1 or paclitaxel alone or in combination and the cell viability was monitored using the flow cytometry (FACS) system. After transfected/treated with indicated agents, cells were collected, and apoptatic cells were measured by propidium iodide and annexin V staining. Early- and late-stage apoptosis were induced by sMEK1 or paclitaxel as well as sMEK1 plus paclitaxel.

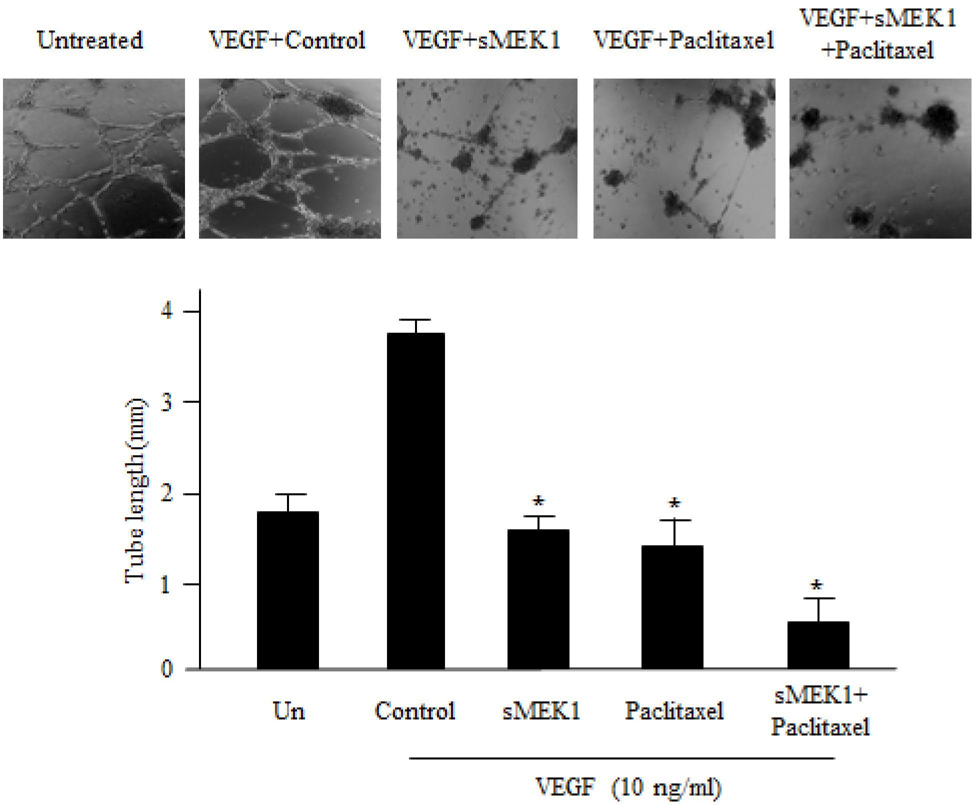


**Fig S2: *In vitro* effect of sMEK1 and paclitaxel on HUVEC tube formation.** Each transfectant was seeded on Growth Factor Reduced Matrigel, and then treated for 48 h with or without VEGF. The quantification of newly formed tubule networks was done from photographs taken using an inverted microscope. The data were expressed as the mean±SD of three independent experiments. *, $P<0.05$ compared to control.
